# To the Editor of the Ceylon Government Gazette

**Published:** 1803-06-01

**Authors:** T. Christie

**Affiliations:** Medical Superintendent General. Columbo


*To the Editor of the Ceylon Government Gazette.*

SIR,

FINDING that the experiment of inoculating with Small-pox matter, patients who have passed through the Cow-pox, has not yet been practised in other parts of India, I request you will state in your next Gazette, that the power of the Vacine disease in preventing the Small-pox has been put to this last test at Jaffnapatnam, where two patients, who had passed through the Cow-pox, were inoculated by Mr. Carnie with Small-pox virus, without any effect being produced.

The patients alluded to, were the children of lieutenant Theil, an intelligent Dutch officer, who with a zeal for truth, and anxiety for the safety of his offspring, highly commendable, desired that they might be submitted to the

 experiment, experiment, in order that he might be perfectly satisfied that his children were completely secured against the effects of the Small-pox, and that the Vaccine disease had lost nothing of its specific qualities in passing through such a variety of persons and climates.

I beg leave to mention this as an example worthy of imitation, and to state that all the medical gentlemen on this island are ready to make the same conclusive experiment on any patients who have passed through the Cow-pox, being perfectly satisfied as to its safety.

I have the honour to be, &c.

*Columbo,* T. CHRISTIE,

*Oct.* 10, 1803. Medical Superintendent General.

